# Evaluation of a task-based community oriented teaching model in family medicine for undergraduate medical students in Iraq

**DOI:** 10.1186/1472-6920-5-31

**Published:** 2005-08-22

**Authors:** Samim A Al-Dabbagh, Waleed G Al-Taee

**Affiliations:** 1 Department of Community Medicine, Mosul College of Medicine, Mosul University, Mosul, Iraq

## Abstract

**Background:**

The inclusion of family medicine in medical school curricula is essential for producing competent general practitioners. The aim of this study is to evaluate a task-based, community oriented teaching model of family medicine for undergraduate students in Iraqi medical schools.

**Methods:**

An innovative training model in family medicine was developed based upon tasks regularly performed by family physicians providing health care services at the Primary Health Care Centre (PHCC) in Mosul, Iraq. Participants were medical students enrolled in their final clinical year. Students were assigned to one of two groups. The implementation group (28 students) was exposed to the experimental model and the control group (56 students) received the standard teaching curriculum. The study took place at the Mosul College of Medicine and at the Al-Hadba PHCC in Mosul, Iraq, during the academic year 1999–2000. Pre- and post-exposure evaluations comparing the intervention group with the control group were conducted using a variety of assessment tools.

**Results:**

The primary endpoints were improvement in knowledge of family medicine and development of essential performance skills. Results showed that the implementation group experienced a significant increase in knowledge and performance skills after exposure to the model and in comparison with the control group. Assessment of the model by participating students revealed a high degree of satisfaction with the planning, organization, and implementation of the intervention activities. Students also highly rated the relevancy of the intervention for future work.

**Conclusion:**

A model on PHCC training in family medicine is essential for all Iraqi medical schools. The model is to be implemented by various relevant departments until Departments of Family medicine are established.

## Background

More than half of newly graduating physicians will be employed as general practitioners (GP). "Family medicine" is almost synonymous with general practice and constitutes a major component of newly graduated doctors' practices. Characteristics of general practice include accessibility, availability, comprehensiveness, maintaining responsibility and long-term doctor/patient relationship [1,2]. Moreover, general practice plays a vital role as an entry point into the health care system, as a link between self-care and professional medical care and substantially influences overall level of care and use of resources in a community [2,3].

Since the Alma-Ata declaration, the concept of Primary Health Care (PHC) has broadened and encompasses both general and family practice, which refer to the specific medical services provided [4]. The PHC team is now recognized as a crucial element in the delivery of community based medical care. A further challenge is to strengthen relationship between the PHC team and other health networks within the community to achieve the Alma-Ata declaration's vision of PHC [5].

Family physicians play a major role in integrating and coordinating care provided to patients and their families. They are responsible for the implementation of the concept of PHC through their work in general practice. Therefore, a well designed and effective training program in family medicine should be an essential component of medical school curricula.

The first medical school in Iraq, Baghdad Medical College, was established in 1927 with the help of Sir Harry Sinderson, who served as its first Dean. The college adopted the Edinbrough curriculum, which reflected standard teaching curricula of the time. Iraq's second medical school, Mosul Medical College, opened in 1959. Other medical colleges were subsequently established throughout Iraq and all adopted the teaching curriculum of Baghdad Medical College, supported by the Ministry of Higher Education (MOHE), although there were no financial or political reasons to adopt the traditional curriculum. In recent years, suggestions have been put forth to adopt innovative or community based curricula, but such attempts have not received the support of clinicians in medical colleges. In 1989, the MOHE opened a new medical college in Tikrit with an innovative curriculum, supported by the World Health Organization. Despite the WHO imprimatur, a shortage of staff led to incomplete implementation of the innovative teaching model and the result was a combination of the two curricula.

Iraqi medical education is a six year programme. The first three years are devoted to basic sciences and the subsequent three years focus on clinical aspects of medical care. The final year is provides hospital based clinical training in medicine (12 weeks), surgery (12 weeks), pediatrics (10 weeks), gynecology and obstetrics (8 weeks) and community medicine (2 weeks).

There is no specified department for teaching family medicine in any of the Iraqi medical colleges, including that of Tikrit. The curricula of all medical schools in Iraq ignore the concept family medicine as a separate entity. Moreover the clinical teaching component is almost entirely hospital-based; students are not exposed to the presentation of diseases outside of a hospital setting and they are often unprepared for the complexity of general practice [6].

Community-based clinical practice is unique in that it provides real-world scenarios as well as a wide range of learning opportunities [7]. Numerous medical schools have adopted the concept of family medicine to better prepare students for the complex eventualities of general practice. The Arabian Gulf University in Bahrain, for example, has established a separate department for Family and Community Medicine. The Family Medicine Clerkship is mandatory final rotation that students must complete prior to sitting for the final qualifying examination. Another model is the United States, where 95 (out of 127) medical schools have separate departments for family medicine and/or PHC, where students spend an average of 5.7 weeks in clinical clerkships in family/community medicine during their final two academic years [8,9].

Medical educators are now focusing on the relevancy of the medical curriculum to the actual health needs of the community and on the ability of newly graduated doctors to solving common health problems. In another words, medical colleges have made significant efforts to produce competent family physicians [10,11].

Traditional medical colleges have also attempted to apply family medicine to their curriculum by expanding their curricula to include (a) multi-disciplinary approaches (b) the scientific rationale for medicine, (c) problem-solving approaches, (d) strengthening understanding of socio-cultural factors, (e) relating the curriculum to the students' needs, and (f) integrating premedical with pre-clinical medical sciences. Thus, community-oriented medical education seems to be the most progressive and effective way of introducing family medicine into the medical curriculum.

The World Health Organization has encouraged all countries to undertake activities to reform medical education and medical practice with a view to increasing relevance, quality of care, cost-effectiveness and equity in health care. In Iraq, there have been a few studies that have reviewed the teaching curriculum of medical schools during the last few years with the aim of developing a national curriculum for medical colleges with relevance to community needs [12].

The undergraduate teaching of family medicine is not a separate entity in the curriculum of Iraqi Colleges of Medicine and the Ministry of Health programs in the delivery of PHC are rely upon active general family practitioners for success. To achieve this goal, a task-based community oriented model in family medicine for Iraqi medical schools was developed.

The aim of the model was to prepare the future graduates for general practice. The main specific objectives were to train undergraduate medical students to:

• demonstrate familiarity with common health problems and their related health traditions

• provide diagnostic, therapeutic and preventive services

• develop physical, social and psychological relationships with families

• promote the general health status of individuals and their families

• activate and encourage team work in health institutions

• advocate for community participation through regional PHC committees

• use research to assess process of family medicine development

## Methods

The study was conducted during the academic year 1999–2000. The methodology of the study was based on a newly proposed job description for general practitioners in Iraq, which combined the activities and responsibilities of GPs with knowledge gained in medical schools and the traditions and customs of the society [13]. The plan was to utilize the tasks and subtasks of the proposed job description to design a task-based health care oriented training model in family medicine for undergraduate medical students. The steps used in the development of the model were the following.

1. Tasks were first grouped under convenience headings and then systematically clustered into four main groups: child health services, maternal health services, common health problems and administration health care activities.

2. A training course programme at a PHCC setting was planned. Learning activities were implemented according to time and place with respect to the tasks and subtasks.

3. Plans for implementing daily activities throughout the course program were outlined. This was done to maximize the effect of the planned training course. The purpose was to create a learning experience that resembled, as much as possible, actual primary care through the introduction of multi-topic health care services. The following activities were conducted daily during the programme:

a. Reviewing the context of learning activities with regards to time, place, resources and students

b. Writing down definite learning objectives

c. Determining critical learning strategies

d. Continuous assessment

4. A sample of medical students was selected for participating in the study. Two groups of students were selected out of eight groups of medical students in their final year at Mosul College of Medicine (28–30 students comprise each administrative group). One group (28 students) was chosen to receive the experimental model (the experiment group). These students had experienced a short gap between their clinical training in the departments of community medicine, pediatrics, and gynecology and obstetric. The comparison group was composed of 2 groups (56 students) who completed their training soon after the experimental group completed their studies.

The duration of implementation of the model was four weeks of block training: two weeks of community medicine, one week of pediatrics and one week of gynecology and obstetrics. Students in the control group following their usual training. The program was to be conducted during the last semester of the academic year, when both groups have finished there usual training in three specialist departments, other than surgery.

Both authors were teaching community medicine in Al-Hadba PHCC. Other tutors were from the staff of community medicine, including psychiatrists and registrars from the departments of pediatrics, gynecology and obstetrics. Tutors were given a one week training course to illustrate the procedures of teaching and evaluation, including seminars and role play. Feed-back from the tutor training was used to modify some of the procedures of implementation of the model.

The setting for the model was the Al-Hadbaa PHCC and the delivery room in the Al-Batool teaching hospital, which is adjacent to the PHCC. Each student completed a log book for all cases and topics of the medical services involved. A pretest evaluation of the intervention group was conducted at the start of the study. A post-training evaluation was conducted for students on the experimental group, as well as for the comparison group using the same roles and criteria.

Different assessment tools were used in this study. For pre- and post-test evaluations, modified essay questions, MCQ's, case management exercises, flowcharts and oral examinations were used. A daily flowchart was given to each student to be completed as homework. The tools were specially designed forms comprising check lists and rating scales to be filled in by the investigators using direct observation. Finally, a 25 item questionnaire was submitted to the 28 trainee students in the intervention group at the end of the training course to solicit their feed-back. Questions referred to the planning of the program, relevance and utility of the working methods, method of running the program and organization attitudes, timing for activities, benefits gained and participants and program evaluation tools used.

A five point rating scale for task analysis was used (0 = not done, 1 = unsatisfactory, 2 = equivocal, 3 = satisfactory, 4 = very satisfactory). Coefficients for the 5 point rating scale were 1, 2, 3, 4 and 5. The score of each item on a question form was expressed as percentage by Guilbert formula [14]:



Where 20 = 100 divided by the maximum coefficient 5



Items related to one variable were grouped together when comparison was needed between variables consisting of many items. The score of each variable is the aggregate score of its components.

## Results

Table 1 illustrates the teaching methods, assessment tools, place and duration of the task-based community oriented teaching model in family medicine for medical students. The model is designed to be implemented over four weeks for final year medical students. The setting should be a well equipped and organized PHCC. The tasks and learning objectives of the four principle functions of the model are illustrated in tables 2, 3, 4, 5.

**Table 1:** Task-based community care oriented teaching model in family medicine

teaching	theory	Classroom lectures, tutorials, discussion groups, procedure book, problem-based learning and workshops
	practical	Small groups discussion, observed field work, supervised field work, log book, self study activities
aids		Growth chart, handouts, posters, flipcharts, video films, blackboard, overhead projector
assessment toots		Log book, problem solving exercises, OSCE, patient management problems, MCQs, Short assays, checklist and rating scale, evaluation of reports and flowcharts
place		Primary health care centre
day and time hours		Saturday through Thursday 8–12 am
duration		one week in each department for each function

**Table 2:** Function: administrative health care Department: community medicine

**principle tasks**	**learning objectives**
- Health educational guiding advice propagation.	- Prepare a health educational topic, identify opportunities for health education during routine clinical work, initiate health talk and use a suitable communication method to clarify critical health educational subjects.
- Community diagnosis through morbidity and mortality reporting.	- Choose simple practical methods for vital events reporting, classify data according to scientific basis, utilize data for diagnosis of community health problems and health needs.
- Surveillance of disease.	- Collect, analyze & distribute data to those responsible.

**Table 3:** Function: child health care department: pediatric

**principle tasks**	**learning objectives**
- Child Nutrition growth & development	- Trace child's growth and monitor feeding habits.
- Breast feeding	- Facilitate an exclusive breast feeding practice during the 1^st ^4–6 months; explain its benefits and when to start giving weaning food.
- ARI control and the rational use of antibiotics	- Identify dangerous criteria and the situations of using specific antibodies.
- Diarrhoeal Disease control and the use of Oral rehydration	- Diagnose diarrhoeal diseases and assess dehydration. Decide which treatment plan is indicated and explain the value, and method of preparing ORS and its alternatives.
- Child protection from dangerous communicable diseases immunization	- Identify type and time of giving vaccines, its contraindications and side effects, its storage, site of giving vaccine and perform properly the national schedule of vaccination to a maximum coverage-rate.
- Management of other common health problems and its related health customs and traditions	- Take a brief history and conduct essential clinical examinations, do specific investigations, diagnose and manage the main health problem and communicate well to manipulate the related health customs or traditions.
- Risky child-diagnosis & follow up	- Pick dangerous specific criteria, diagnose risky child, prescribe essential method of management, make a follow up schedule, and monitor indicators of improvement
- Health-educational advice propagation	- Detect the actual health educational problem through proper communication, determine the essential educational needs, suggest a proper way and timing for introducing the advice.
- Screening for rheumatic heart diseases.	- Auscultate pupil's heart carefully, detect any added sounds or murmur, examine the tonsils and relate the findings to past history of recurrent attacks of tonsillitis.
- Essential measures to obtain a healthy eye and vision.	- Examine the eyelids and eyeball carefully, identify any disturbed vision, detect critical or risky criteria, diagnose the problem and deal with it accordingly.
- Referral services.	- Determine indications for referral, including method and direction and follow up referred cases.
- Prevention and control of any outbreak of epidemic among pupils.	- Notify about the case, isolate it by a suitable method, give essential treatment and sick leave needed, examine contacts, put under observation risky ones and provide essential health protective measures.

**Table 4:** Function: maternal health care department: obstetric and gynecology

**principle tasks**	**learning objectives**
- Pre-marital & health-care services.	- Take proper family, medical history, educate couples about the essentials, order for CXR, blood group, R.H & other investigations for STD. - Take proper obstetric history, conduct properly clinical examination searching for signs of anemia hypertension, edema, urinary tract disorders, pelvic size and shape, and any fetal abnormality, do a schedule for antenatal visit and weight checking giving antitetanus toxoid at the proper time & calculate the expected date for delivery.
- Management of common gestational health problems.	- Diagnose and prescribe treatment for anemia, hypertension, urinary tract infections, toxoplasmosis and diabetes, and follow up patients through out gestational period.
Risky pregnancies – diagnosis and follow up.	- Detect and manage risky pregnancies and follow them up.
Delivery-health-care services.	- Facilitate process of delivery to be normal uncomplicated vaginal labor and promote uterine contractions for expulsion of a head presented fetus under sterile conditions.
Essential postnatal health care services.	- Examine for unevoluted uterus, bleeding & promote healthy purperium, lactation and look for breast complications including over encouragement or crackled nipples.
- Health educational advice for pregnant and lactating women.	- Use proper methods of communication to explain essential nutrient materials needed and the value of attending MCH clinics periodically, taking tonic drugs, vaccination and care of breasts.
- Immunization of women during the age of child bearing.	- Protect women through proper implementing of vaccination program against German measles and tetanus.
- Referral services.	- Put criteria for risky patient's referral and direction, identify proper time and essential measures for referral, and follow up referred patients.
- Screening for the risk of developing breast carcinoma.	- Perform a proper clinical examination of breasts and detect any lump and manage accordingly specially for those with positive family history of breast cancer.
- Follow up hydatiform-mole	- Monitor women with history of hydatidiform mole, prescribe essential contraceptive, give methotrexate, and follow them up.
- Management of common health problems of lactating breast.	- Diagnose and manage common breast problems e.g. crackled nipples, retracted malformed nipples, and breast abscess.
- Infertility and menstrual cycle regulation measures.	- Take proper menstrual history, detect any abnormality, relate findings to failure of conception, and suggest proper treatment method.
- Family planning services.	- Display a proper action for a better child spacing time, and communicate properly to justify a routine attendance of family planning clinics.

**Table 5:** Function: management of community health problems department: medicine

**principle tasks**	**learning objectives**
- Practicing an evidence based clinical medicine	- Take proper clinical history, conduct general and systemic examination, select essential investigations and perform diagnosis according to specific criteria
- Making provisional and definitive diagnosis.	- Practice putting differential diagnosis and identify the most logical diagnosing criterion
- Prescription and evaluation of treatment.	- Practice writing drugs prescription and explain route and method of administration and its main side effects, and evaluate its impact
- Dealing with current health traditions and customs	- Choose the negative tradition, plan to alter it, use a suitable & effective method of communication.
- Prevention and control of communicable disease conditions	- Display essential measures for preventing and controlling infectious cases (environmental sanitation, active and passive immunization, isolation of cases, tracing of contacts .. etc.
- Health-educational advice	- Propagate essential and primitive primary health care educational guidance (specially in regard to safe water, immunization program, and MCH services).
- Building a positive relationship with families.	- Practice the initiation and continuity of an active relationship with clients and their family
- Notification and referral services of dangerous diseases	- Identify critical cases, practice notification activities, and refer cases according to indication
- Referral of chronic disease	- Put criteria of referral, identify its presence, practice referral measures, and follow up cases to ensure continuity of health care

Table 6 shows the pre- and post-test results of the implementation group and those of the comparison group regarding tested knowledge and skills. There was significant improvement in both areas following the application of the training model. The percentage of those who received a total score >75 increased significantly for all types of examinations conducted in comparison to pretest results and those of the control group.

**Table 6:** Assessment results for knowledge and skills of implementation (pre and post exposure) and control groups.

**assessment tools**		**% of students who got the specified degrees**		
		
		**<50**	**50–75**	**>75**
**multiple choice questions**	**a**	7.14	42.9	50.0
	**b**	0	10.7	89.3
	**c**	8.9	39.3	51.8

**modified assay questions**	**a**	3.6	78.6	17.9
	**b**	3.6	39.3	57.1
	**c**	8.9	66.1	25.0

**case-management exercises**	**a**	25.0	57.1	17.9
	**b**	0	0.0	100.0
	**c**	3.2	58.9	17.9

**flow charts**	**a**	53.6	46.4	0.0
	**b**	0	0.0	100.0
	**c**	55.4	33.9	10.7

**oral examination**	**a**	10.7	75.0	14.3
	**b**	0.0	21.4	78.8
	**c**	21.4	60.7	17.9

**grand total**	**a**	20.0	60.0	20.0
	**b**	0.0	3.6	96.4
	**c**	23.6	51.8	24.6

a. Implementation group (28 students) pre training assessment

b. Implementation group (28 students) post training assessment

c. Control group (56 students)

Table 7 reveals the pre- and post-exposure results for students' attitudes towards clinical training at PHCC. The percentages of students who scored a three or a four on attitudes towards learning, response to advice, initiating and sharing ideas rose from 7.2%, 21.4%, 17.9%, 3.6% to 82.3%, 100.0%, 57.3% and 60.0% respectively after exposure. These rates were also higher than the relevant rates among the control group which were 7.1%, 19.6%, 14.3% and 5.4% respectively.

**Table 7:** Assessment results of some attitude parameters of implementation (pre and post exposure) and control groups

**attitude's parameters**		**% of students who got the specified scores**			
		
		**0–1**	**1–2**	**2–3**	**3–4**
**attitude toward learning**	**a**	91.0	0	1.8	7.2
	**b**	10.7	0	7.0	82.3
	**c**	76.8	12.5	3.6	7.1

**response to advice by tutors**	**a**	35.7	42.9	0	21.4
	**b**	0	0	0	100.0
	**c**	30.4	41.1	9.0	19.6

**initiatives by studies**	**a**	67.9	0	35.7	17.9
	**b**	7.0	0	35.7	57.3
	**c**	64.3	5.4	16.1	14.3

**sharing ideas within the group and with tutors**	**a**	60.7	25.0	21.4	3.6
	**b**	7.0	10.7	21.4	60.9
	**c**	58.9	28.6	7.1	5.4

a. Implementation group (28 students) pre training assessment

b. Implementation group (28 students) post training assessment

c. Control group (56 students)

Table 8 shows that communication skills were poor among students before the application of the training model and increased to a satisfactory level following the intervention training. Table 4 also reveals that there was little improvement in those skills in the control groups after they completed their classical training in the specified departments.

**Table 8:** Assessment results for communication skills with patients at PHCC among implementation (per and post exposure) and control groups.

**Communication parameters**	**% of students show proper communication**		
	
	**a**	**b**	**c**
are appropriate visual methods used?	21.4	89.3	23.2
is the communication brief?	43.0	85.7	46.4
is the communication unhurried?	18.0	96.4	16.1
are the facts accurate?	82.0	100.0	71.4
is the argument logical and clearly structured?	43.0	78.6	44.6
is enough detail provided?	29.0	96.4	25.0
are familiar words used?	29.0	75.0	28.6
is the sentence structure simple?	25.0	100.0	28.6
is the patient greeted?	29.0	26.4	30.4
is the patient spoken to by name?	0.0	78.6	10.7
is the patient existing knowledge explored?	0.0	64.3	5.4
are the patient's beliefs respected?	68.0	100.0	69.6
is he credited for appropriate action?	64.8	85.7	60.7
are blame and condemnation avoided?	29.0	100.0	33.9
is concern shown for the patient's problem?	18.0	100.0	25.0
dose any solution offered actually solved the problem as seen by the patient?	32.0	89.3	35.7
is the patient asked to apply information?	3.6	85.7	10.7
are the patient knowledge & understanding tested?	0.0	96.4	8.9

a. Implementation group (28 students) pre training assessment

b. Implementation group (28 students) post training assessment

c. Control group (56 students)

Significant improvement in the skills of measuring arterial blood pressure and preparing a blood film for malaria among the implementation group after the one month training for family medicine at the PHCC were also observed (Tables 9 and 10).

**Table 9:** Assessment result for student's skills on measuring arterial blood pressure.

**component task**		**% of students who got the specified rating scores**				
		
		**0**	**1**	**2**	**3**	**4**
explaining what will be done	a	89.3	10.7	0	0	0
	b	0	0	3.6	3.6	92.8

explaining the procedure in patient language	a	53.6	35.7	10.7	0	0
	b	0	0	7.1	21.4	71.4

checking the cuff size	a	57.1	21.4	17.9	3.6	0
	b	0	0	3.6	3.6	92.8

rolling up sleeve	a	35.7	35.7	17.9	0	10.7
	b	0	0	0	17.9	82.1

centering the cuff bladder over brachial artery	a	0	0	3.6	7.1	89.3
	b	0	0	0	0	100.0

positioning & supporting the arm at heart level	a	53.6	21.4	7.1	17.8	0
	b	0	0	10.7	10.7	78.6

taking palpation	a	100.0	0	0	0	0
	b	0	0	7.1	10.7	82.0

waiting 30 seconds to allow the arm to rest	a	100.0	0	0	0	0
	b	0	0	7.1	0	92.9

repositioning of the arm at heart level	a	53.6	17.9	10.7	8.9	8.8
	b	0	0	0	3.6	96.4

placing the diaphragm over brachial artery	a	0	0	0	0	100.0
	b	0	0	0	0	100.0

inflating the cuff 20 mm above palpatory artery	a	58.9	41.1	0	0	0
	b	0	0	10.8	7.1	82.1

recording Bd. P	a	0	0	0	0	100.0
	b	0	0	0	0	100.0

replacing the arm at rest	a	55.4	23.2	10.7	101	0
	b	0	0	0	17.9	82.1

offering patient time to ask questions	a	100.0	0	0	0	0
	b	0	0	25.0	0	75.0

a. Implementation group (28 students) pre training assessment

b. Implementation group (28 students) post training assessment

**Table 10:** Assessment result for student's skills on making a blood film for malaria.

**Procedure/component task**		**% of students got the specified rating score**				
		
		**0**	**1**	**2**	**3**	**4**
clearing the slide	a	89.3	3.6	5.4	1.7	0
	b	0	0	7.1	10.7	82.2

positioning the drop one cm from slide end	a	53.6	10.7	35.7	0	0
	b	0	0	0	10.7	89.3

using a cut edge slide as spreader	a	35.7	17.9	23.2	5.4	17.8
	b	0	3.6	3.6	35.7	57.1

moving hand firmly and steadily	a	58.9	23.2	0	10.7	7.2
	b	0	0	0	7.1	92.9

making thin blood smear	a	35.7	17.9	17.9	26. 8	1.7
	b	0	0	0	42.9	57.1

shaking blood Smear in air to dry	a	53.6	35.7	0	1.8	8.9
	b	0	0	0	32.1	67.9

labeling the patient's name on slide	a	73.2	5.4	8.9	12.5	0
	b	3.6	0	0	0	96.4

making 2 slides per patient	a	82.1	0	0	0	17.9
	b	3.6	0	0	0	96.4

putting dry slide upright on slide	a	53.6	17.9	10.7	17.8	0
	b	0	0	7.1	35.7	57.2

a. Implementation group (28 students) pre training assessment

b. Implementation group (28 students) post training assessment

Finally, evaluation of the training model by the 28 trainee students revealed a satisfaction index ranging from 91.4–100.0% (Table 11).

**Table 11:** Evaluation of the training model by trainee students using 25 questions items.

**evaluation aspects**	**total scores**	**satisfaction index**
planning (4 questions)	135.5	96.8%
relevance and utility (5 questions)	136.8	97.7%
program's running & organizer's attitudes (5 questions)	136.8	97.7%
activities according to available time (4 questions)	137.0	97.9%
benefits gained by trainees (4 questions)	135.8	97.0%
model evaluations (3 questions)	140.0	100.0%

## Discussion

A task-based community oriented teaching model in family medicine was developed and tested on final year medical students. The study found significant improvement in knowledge and performance skills following exposure to the model.

The study also benefited from the students' enthusiasm. Participating students were eager to learn a greater variety of skills and to examine large number of readily accessible cases. The model is easily applicable at little or no extra cost to standard curricula.

Nevertheless, acceptance of the programme, in the long run, would be improved if tutors from all clinical departments participate in the training model. As the Al-Hadba PHCC is designed for training students, precautions should be taken to reduce the burden on staff if the program is to be implemented in other PHCCs.

The staff evaluation was not blind and was done primarily by community medicine tutors who might be biased towards such a program. This may partially affect the results of the evaluation. Nonetheless, answers to the evaluation questions were pre-determined and were standardized from books and practices by academic staff from different departments to decrease the possibility of such biases.

The teaching of family medicine should be considered for inclusion in the curriculum of Iraqi medical schools. Not only are the majority of newly graduated doctors involved in general practice but there strong reasons for arguing that family medicine should be the core setting for medical training. Most medical schools now have separate departments of family medicine. These departments are responsible for teaching and training students with a curriculum which is frequently updated according to health needs and priorities [15,16].

Medical school students should be exposed to a broad range of educational experiences and gain practical skills. They also should be given time to communicate with their patients to allow them sufficient time to develop an understanding of their problems. Issues relating to accessibility, quality of care and cost can be easily addressed in a PHCC setting where the majority of medical care activities occur. In addition, it has been found that patients at the PHCC both enjoy taking part in undergraduate training and receive greater benefits from the process [17,18].

A task-based community oriented training model was developed. The method integrated PHC services with the tasks of family physicians; this combination helped demonstrate the skills that need to be taught to undergraduate medical students. Analysis of relevant and priority medical services was used to formulated the model. The model is believed to cover all of the required items suggested by similar programs in other studies[19,20]. Community oriented primary health care has consistently showed dramatic positive changes in the health status of the population in several countries. To achieve this goal, obstacles should be overcome so that effective training can be implemented within the traditional medical school curriculum [21].

The principle characteristic that distinguishes the task-based health-care oriented teaching model advocated by the present study is the method by which the skills were identified, using appropriate instructional and assessment methods. The sequence of stages was initiated by obtaining detailed information about the nature of a family physician's job at PHC. This was followed by a break-down of those tasks into subtasks, which were then incorporated into the training model.

The teaching model for undergraduates' clinical training in family medicine relies heavily on the multi-disciplinary nature of instructional strategies. Those which are practiced frequently on a daily basis include: plenary and small group discussions, case management exercises, flow charts, observed and supervised field work, in addition to the teaching and practice of certain essential performance skills. These strategies have enhanced the learning process through student centered approaches which encourage an active learning. This differs from current instructional strategies, which are teacher-centered and often result in passive learning. Students' participation in the learning process of PHC will produce versatile doctors who will be better equipped to cope with constant changes. Moreover, implementation of the training model promoted self-directed teamwork learning, with its significant effect on improving medical education. These structured learning experiences are now believed to constitute a major part in the medical education arena [7,22,23].

Assessment of the trainees and their follow up throughout the course program is similar to that reported in other studies [24,25]. The structured learning experiences are much more likely to change students knowledge, attitude and lead to the development of proper clinical skills.

The model should be further reviewed according to the changing health needs of the community. Reforms and continuous evaluations are also essential for monitoring the model and improving utilization of health services. Finally, better results expected if the model were applied by a department of family medicine, which should be established in each medical school [26,27].

Ironically, in Iraq, emphasis has been placed on family medicine as a specialty for newly graduated doctors although no training program exists for undergraduates. In developed countries, it has been suggested that emphasis on family medicine reversed the decline in general practice and high expectations are now placed on family medicine departments to solve pressing health care system problems [28-30]. In the United States, for example, suggestions have been put forth to design a comprehensive family medicine career development program encompassing elementary through postgraduate education. The aim will be to identify youth who wish to become future family physicians [31,32]. Other models of teaching family medicine include teaching the entire curriculum in the community. Such an approach might be less effective in Iraq than a faculty-based model based on the college's curriculum and supervised by the department of family medicine.

## Conclusion

A significant deficiency in knowledge and skills has been observed among students who were not trained for family medicine in a PHCC setting. It is therefore recommended to implement a task-based community oriented model for teaching family medicine in all Iraqi medical schools. Training should be supervised by clinical departments until a separate department of family medicine is established.

## Competing interests

The author(s) declare that they have no competing interests.

## Authors' contributions

Both authors contributed equally in the designing, implementation of the model, analyzing data, and drafting the manuscript. Both authors read and approved the final manuscript.

## Pre-publication history

The pre-publication history for this paper can be accessed here:


